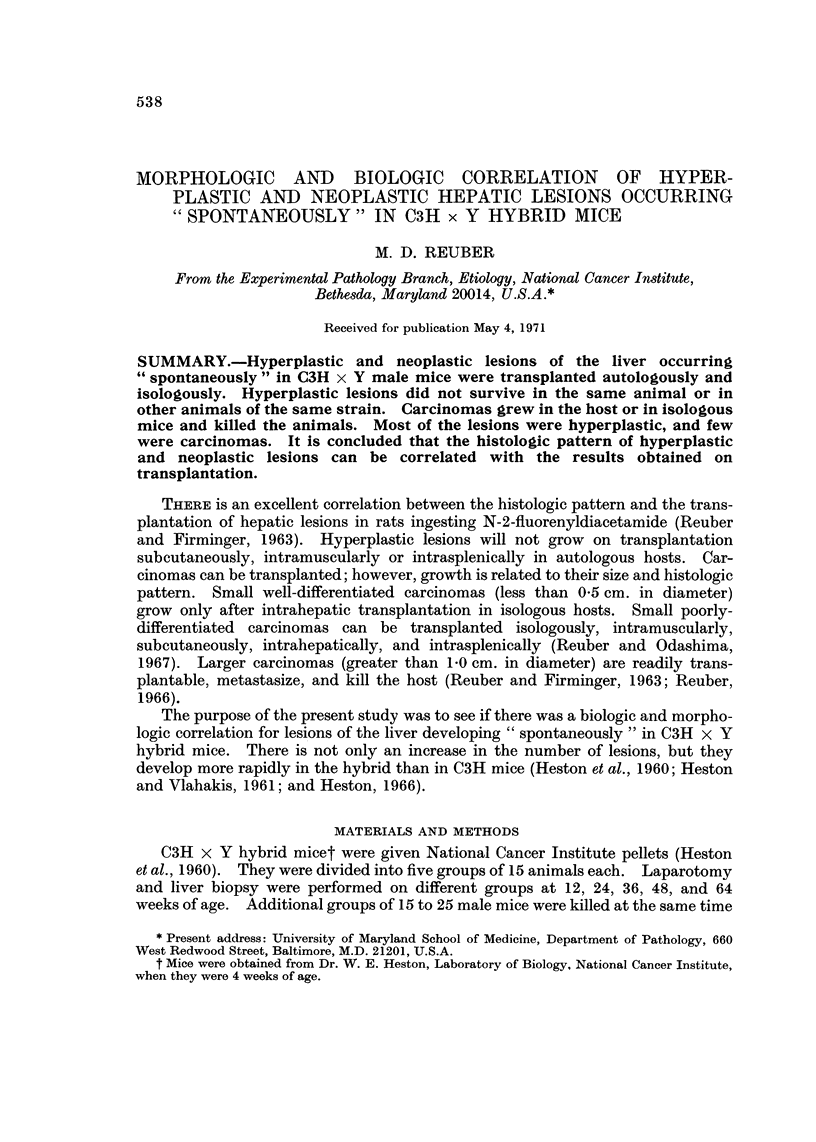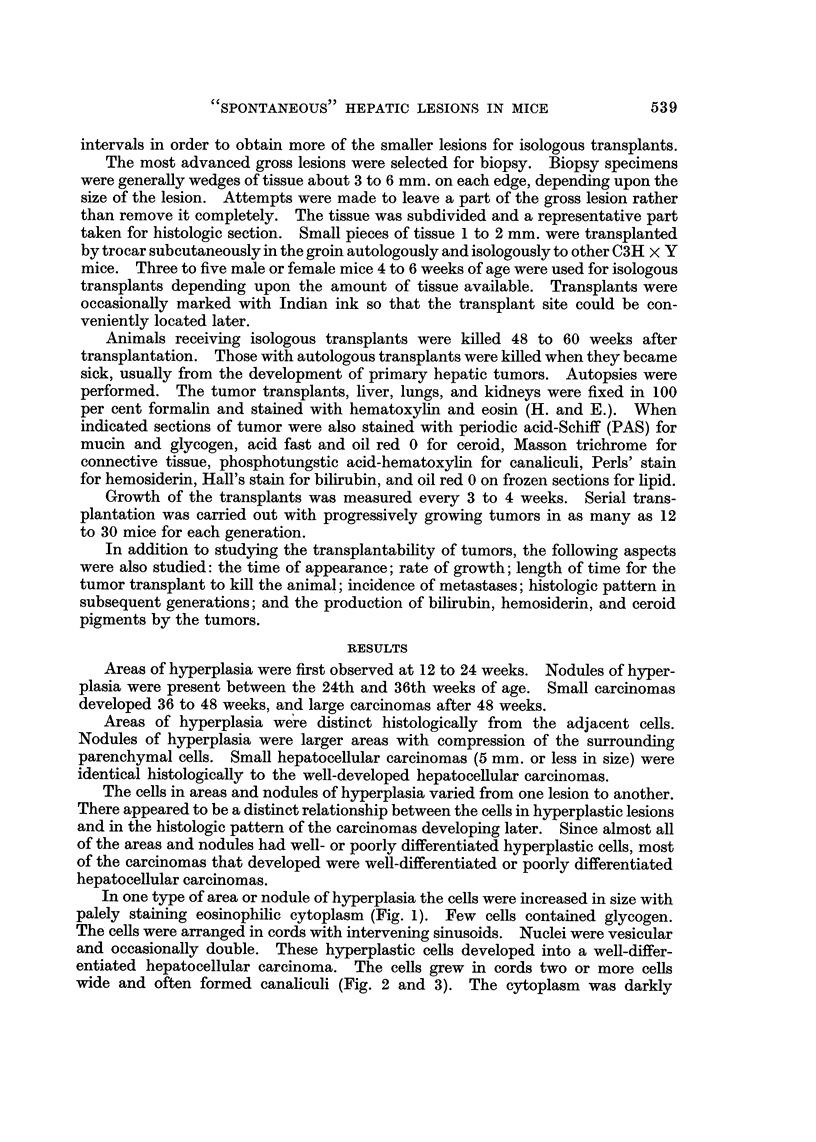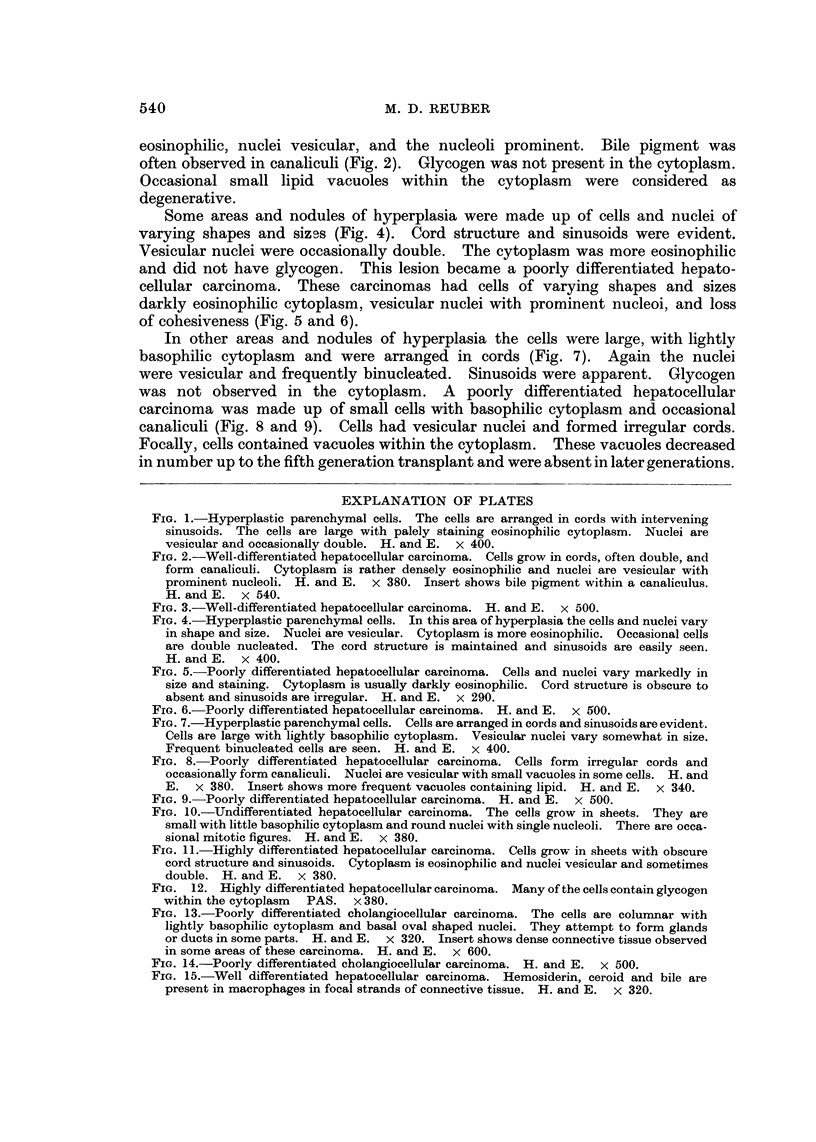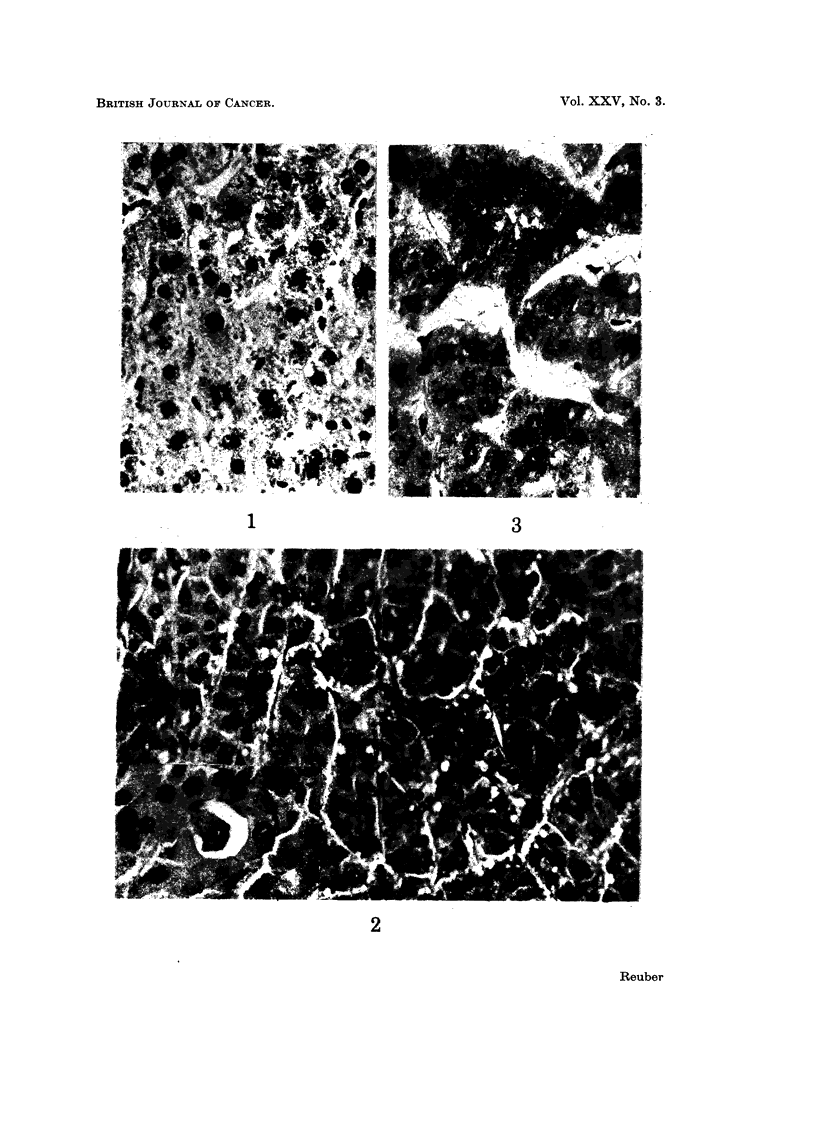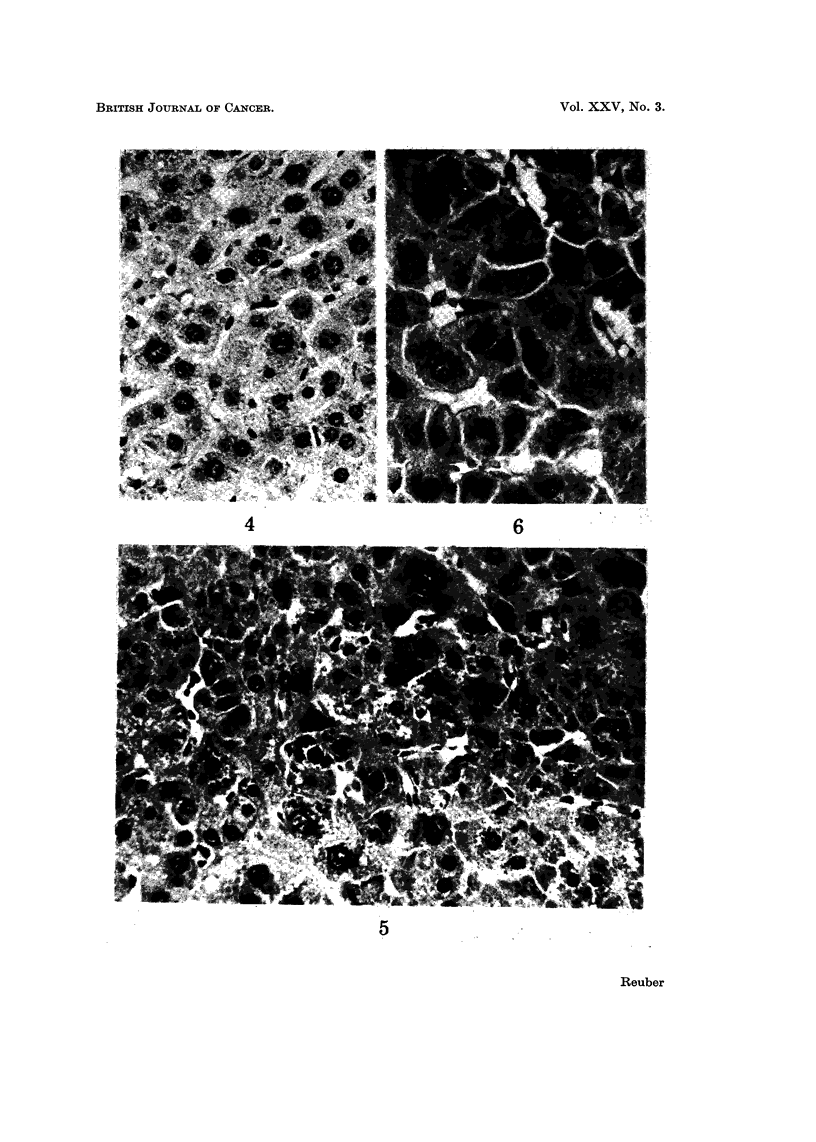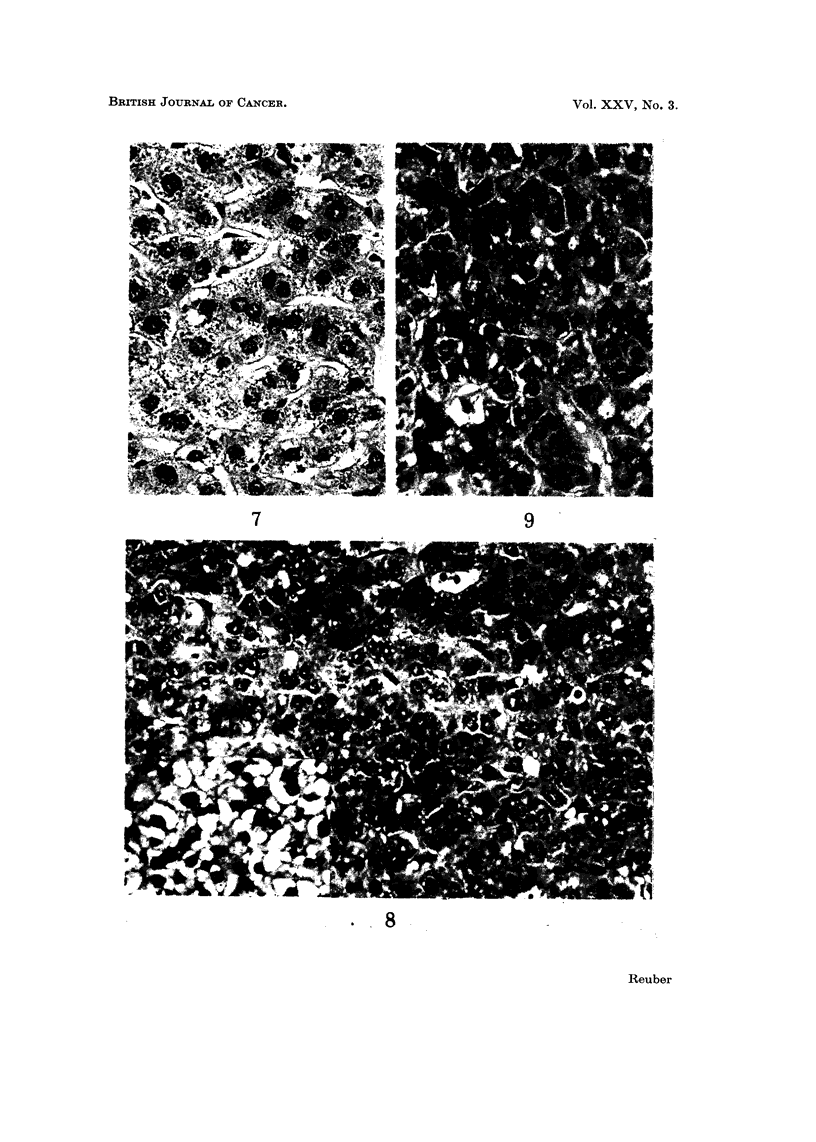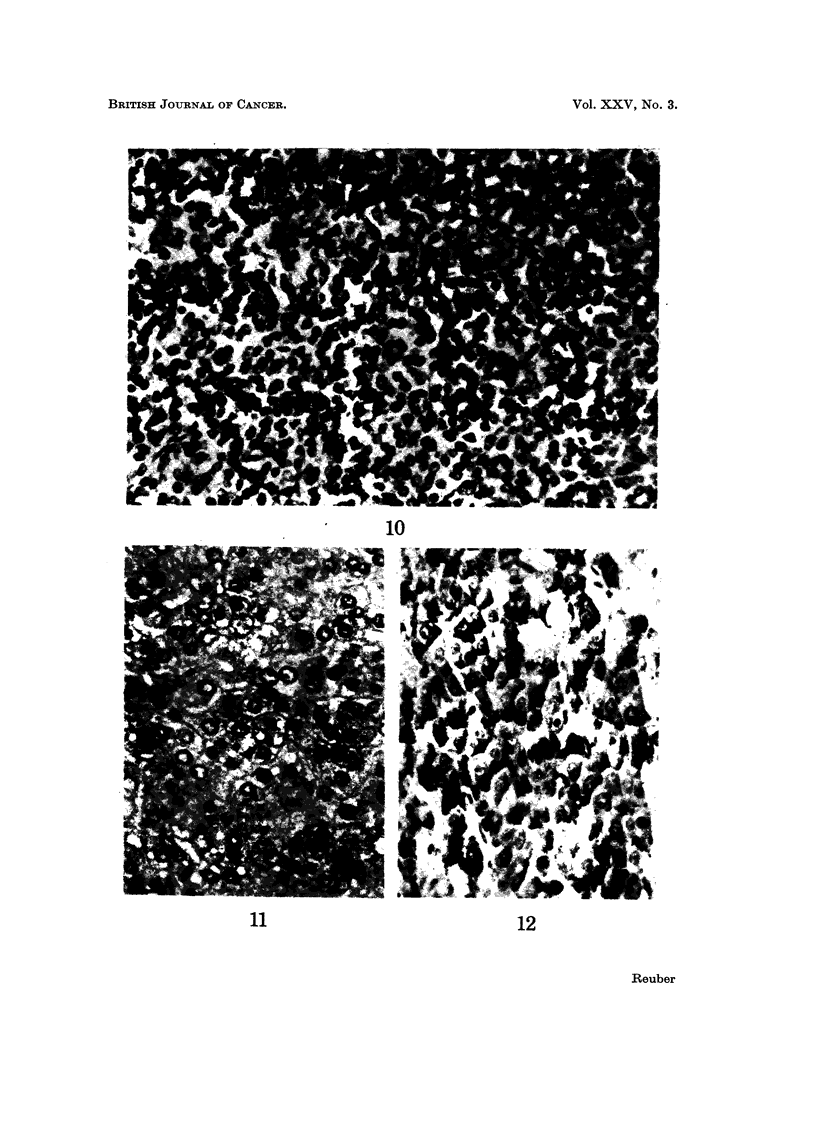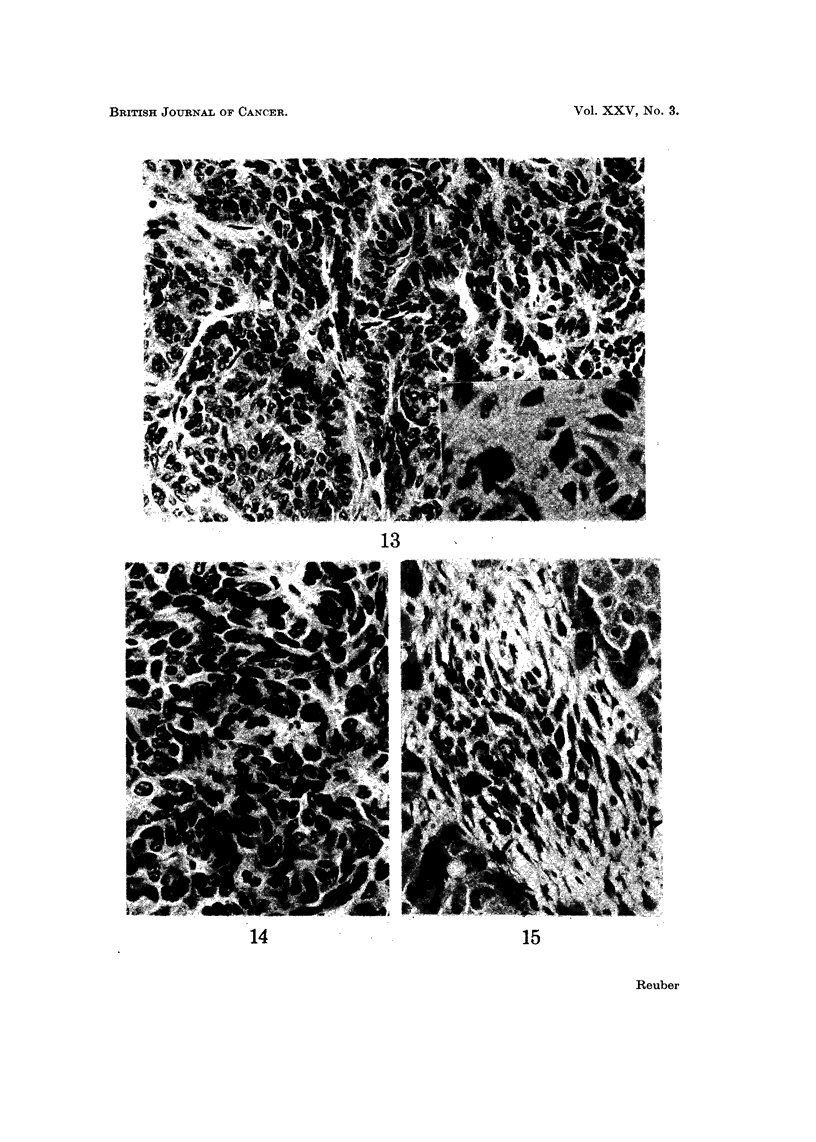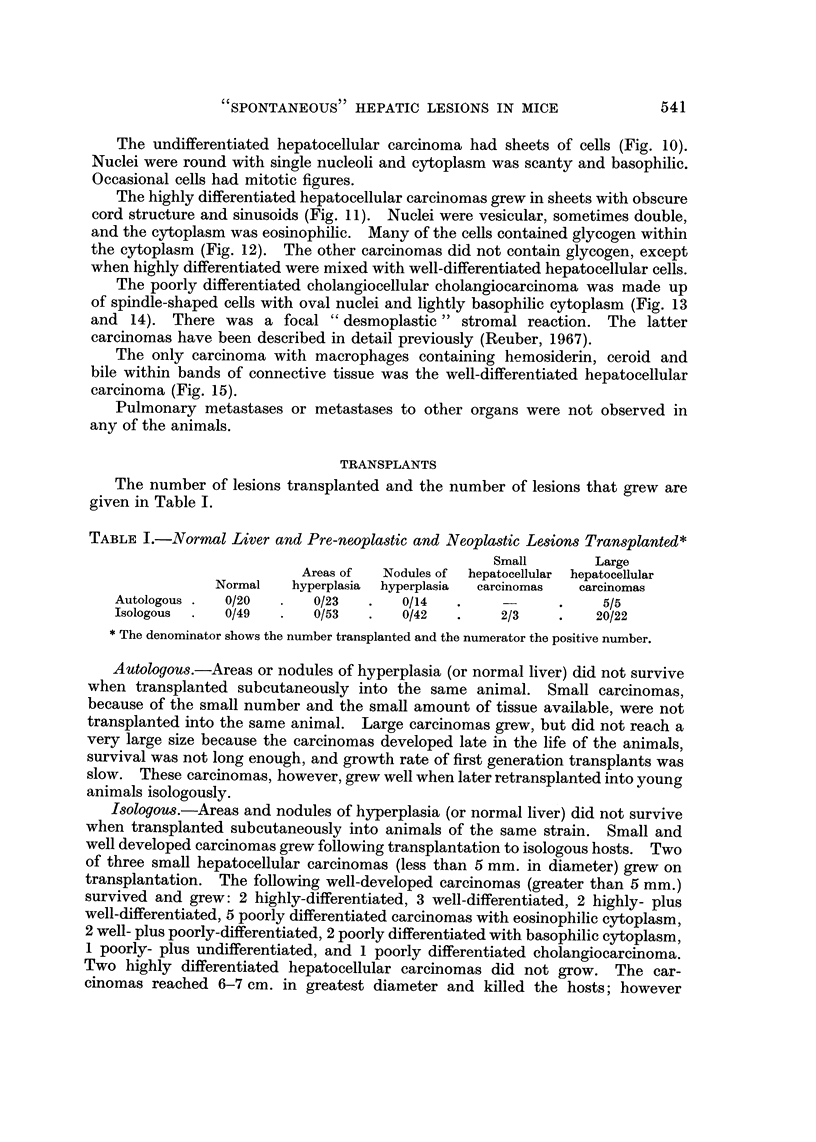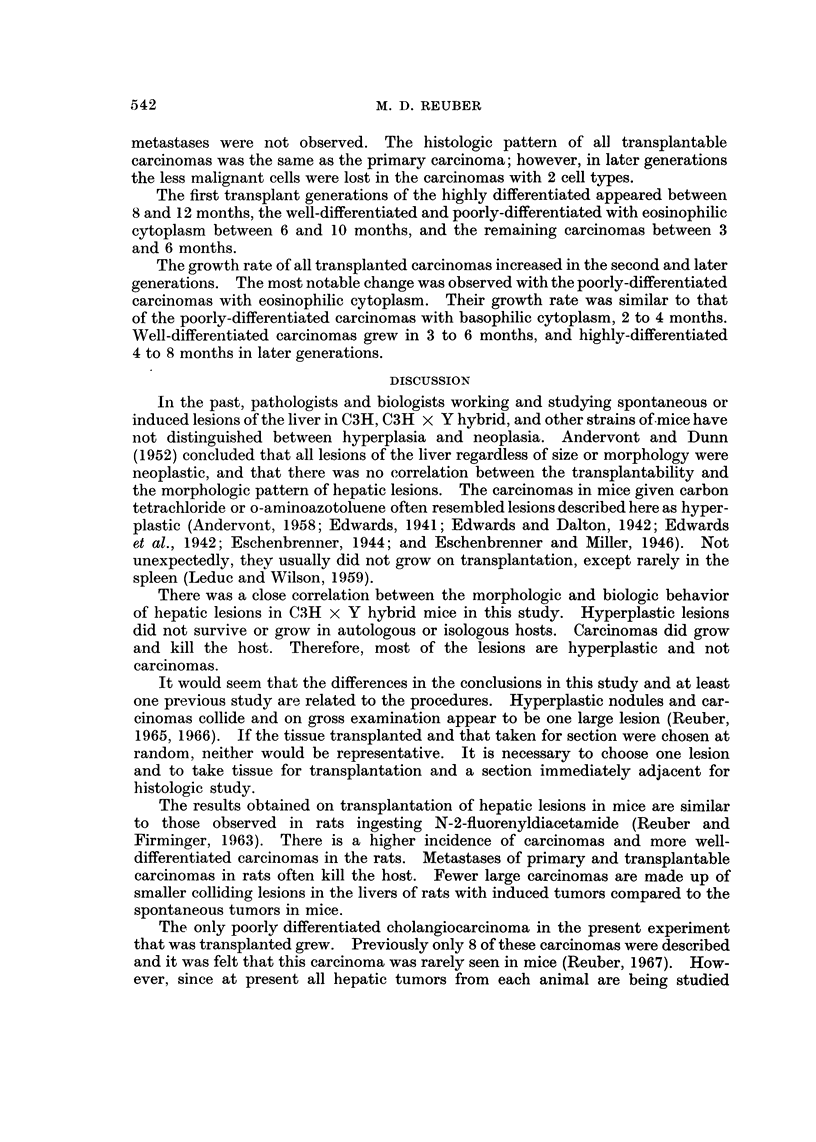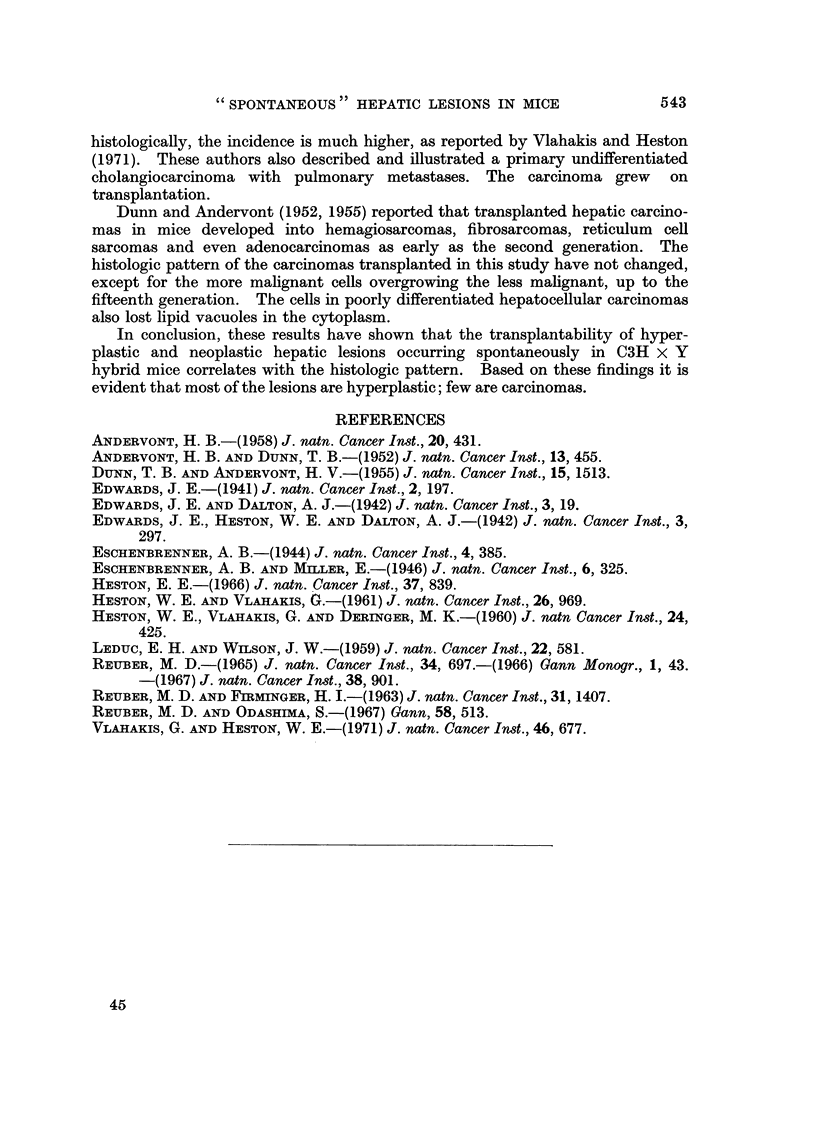# Morphologic and Biologic Correlation of Hyperplastic and Neoplastic Hepatic Lesions Occurring “Spontaneously” in C3H × Y Hybrid Mice

**DOI:** 10.1038/bjc.1971.69

**Published:** 1971-09

**Authors:** M. D. Reuber

## Abstract

**Images:**


### MORPHOLOGIC AND BIOLOGIC CORRELATION OF HYPERPLASTIC AND NEOPLASTIC HEPATIC LESIONS OCCURRING "SPONTANEOUSLY" IN C3H × Y HYBRID MICE

M. D. REUBER

*From the Experimental Pathology Branch, Etiology, National Cancer Institute, Bethesda, Maryland* 20014, *U.S.A.**

Received for publication May 4, 1971

**SUMMARY.—Hyperplastic and neoplastic lesions of the liver occurring "spontaneously" in C3H × Y male mice were transplanted autologously and isologously. Hyperplastic lesions did not survive in the same animal or in other animals of the same strain. Carcinomas grew in the host or in isologous mice and killed the animals. Most of the lesions were hyperplastic, and few were carcinomas. It is concluded that the histologic pattern of hyperplastic and neoplastic lesions can be correlated with the results obtained on transplantation.**

THERE is an excellent correlation between the histologic pattern and the transplantation of hepatic lesions in rats ingesting N-2-fluorenyldiacetamide (Reuber and Firminger, 1963). Hyperplastic lesions will not grow on transplantation subcutaneously, intramuscularly or intrasplenically in autologous hosts. Carcinomas can be transplanted; however, growth is related to their size and histologic pattern. Small well-differentiated carcinomas (less than 0·5 cm. in diameter) grow only after intrahepatic transplantation in isologous hosts. Small poorly-differentiated carcinomas can be transplanted isologously, intramuscularly, subcutaneously, intrahepatically, and intrasplenically (Reuber and Odashima, 1967). Larger carcinomas (greater than 1·0 cm. in diameter) are readily transplantable, metastasize, and kill the host (Reuber and Firminger, 1963; Reuber, 1966).

The purpose of the present study was to see if there was a biologic and morphologic correlation for lesions of the liver developing "spontaneously" in C3H × Y hybrid mice. There is not only an increase in the number of lesions, but they develop more rapidly in the hybrid than in C3H mice (Heston *et al.*, 1960; Heston and Vlahakis, 1961; and Heston, 1966).

### MATERIALS AND METHODS

C3H × Y hybrid mice† were given National Cancer Institute pellets (Heston *et al.*, 1960). They were divided into five groups of 15 animals each. Laparotomy and liver biopsy were performed on different groups at 12, 24, 36, 48, and 64 weeks of age. Additional groups of 15 to 25 male mice were killed at the same time

* Present address: University of Maryland School of Medicine, Department of Pathology, 660 West Redwood Street, Baltimore, M.D. 21201, U.S.A.

† Mice were obtained from Dr. W. E. Heston, Laboratory of Biology, National Cancer Institute, when they were 4 weeks of age.

intervals in order to obtain more of the smaller lesions for isologous transplants.

The most advanced gross lesions were selected for biopsy. Biopsy specimens were generally wedges of tissue about 3 to 6 mm. on each edge, depending upon the size of the lesion. Attempts were made to leave a part of the gross lesion rather than remove it completely. The tissue was subdivided and a representative part taken for histologic section. Small pieces of tissue 1 to 2 mm. were transplanted by trocar subcutaneously in the groin autologously and isologously to other C3H × Y mice. Three to five male or female mice 4 to 6 weeks of age were used for isologous transplants depending upon the amount of tissue available. Transplants were occasionally marked with Indian ink so that the transplant site could be conveniently located later.

Animals receiving isologous transplants were killed 48 to 60 weeks after transplantation. Those with autologous transplants were killed when they became sick, usually from the development of primary hepatic tumors. Autopsies were performed. The tumor transplants, liver, lungs, and kidneys were fixed in 100 per cent formalin and stained with hematoxylin and eosin (H. and E.). When indicated sections of tumor were also stained with periodic acid-Schiff (PAS) for mucin and glycogen, acid fast and oil red 0 for ceroid, Masson trichrome for connective tissue, phosphotungstic acid-hematoxylin for canaliculi, Perls' stain for hemosiderin, Hall's stain for bilirubin, and oil red 0 on frozen sections for lipid.

Growth of the transplants was measured every 3 to 4 weeks. Serial transplantation was carried out with progressively growing tumors in as many as 12 to 30 mice for each generation.

In addition to studying the transplantability of tumors, the following aspects were also studied: the time of appearance; rate of growth; length of time for the tumor transplant to kill the animal; incidence of metastases; histologic pattern in subsequent generations; and the production of bilirubin, hemosiderin, and ceroid pigments by the tumors.

### RESULTS

Areas of hyperplasia were first observed at 12 to 24 weeks. Nodules of hyperplasia were present between the 24th and 36th weeks of age. Small carcinomas developed 36 to 48 weeks, and large carcinomas after 48 weeks.

Areas of hyperplasia were distinct histologically from the adjacent cells. Nodules of hyperplasia were larger areas with compression of the surrounding parenchymal cells. Small hepatocellular carcinomas (5 mm. or less in size) were identical histologically to the well-developed hepatocellular carcinomas.

The cells in areas and nodules of hyperplasia varied from one lesion to another. There appeared to be a distinct relationship between the cells in hyperplastic lesions and in the histologic pattern of the carcinomas developing later. Since almost all of the areas and nodules had well- or poorly differentiated hyperplastic cells, most of the carcinomas that developed were well-differentiated or poorly differentiated hepatocellular carcinomas.

In one type of area or nodule of hyperplasia the cells were increased in size with palely staining eosinophilic cytoplasm (Fig. 1). Few cells contained glycogen. The cells were arranged in cords with intervening sinusoids. Nuclei were vesicular and occasionally double. These hyperplastic cells developed into a well-differentiated hepatocellular carcinoma. The cells grew in cords two or more cells wide and often formed canaliculi (Fig. 2 and 3). The cytoplasm was darkly eosinophilic, nuclei vesicular, and the nucleoli prominent. Bile pigment was often observed in canaliculi (Fig. 2). Glycogen was not present in the cytoplasm. Occasional small lipid vacuoles within the cytoplasm were considered as degenerative.

Some areas and nodules of hyperplasia were made up of cells and nuclei of varying shapes and sizes (Fig. 4). Cord structure and sinusoids were evident. Vesicular nuclei were occasionally double. The cytoplasm was more eosinophilic and did not have glycogen. This lesion became a poorly differentiated hepatocellular carcinoma. These carcinomas had cells of varying shapes and sizes darkly eosinophilic cytoplasm, vesicular nuclei with prominent nucleoi, and loss of cohesiveness (Fig. 5 and 6).

In other areas and nodules of hyperplasia the cells were large, with lightly basophilic cytoplasm and were arranged in cords (Fig. 7). Again the nuclei were vesicular and frequently binucleated. Sinusoids were apparent. Glycogen was not observed in the cytoplasm. A poorly differentiated hepatocellular carcinoma was made up of small cells with basophilic cytoplasm and occasional canaliculi (Fig. 8 and 9). Cells had vesicular nuclei and formed irregular cords. Focally, cells contained vacuoles within the cytoplasm. These vacuoles decreased in number up to the fifth generation transplant and were absent in later generations.

---

EXPLANATION OF PLATES

FIG. 1.—Hyperplastic parenchymal cells. The cells are arranged in cords with intervening sinusoids. The cells are large with palely staining eosinophilic cytoplasm. Nuclei are vesicular and occasionally double. H. and E. × 400.

FIG. 2.—Well-differentiated hepatocellular carcinoma. Cells grow in cords, often double, and form canaliculi. Cytoplasm is rather densely eosinophilic and nuclei are vesicular with prominent nucleoli. H. and E. × 380. Insert shows bile pigment within a canaliculus. H. and E. × 540.

FIG. 3.—Well-differentiated hepatocellular carcinoma. H. and E. × 500.

FIG. 4.—Hyperplastic parenchymal cells. In this area of hyperplasia the cells and nuclei vary in shape and size. Nuclei are vesicular. Cytoplasm is more eosinophilic. Occasional cells are double nucleated. The cord structure is maintained and sinusoids are easily seen. H. and E. × 400.

FIG. 5.—Poorly differentiated hepatocellular carcinoma. Cells and nuclei vary markedly in size and staining. Cytoplasm is usually darkly eosinophilic. Cord structure is obscure to absent and sinusoids are irregular. H. and E. × 290.

FIG. 6.—Poorly differentiated hepatocellular carcinoma. H. and E. × 500.

FIG. 7.—Hyperplastic parenchymal cells. Cells are arranged in cords and sinusoids are evident. Cells are large with lightly basophilic cytoplasm. Vesicular nuclei vary somewhat in size. Frequent binucleated cells are seen. H. and E. × 400.

FIG. 8.—Poorly differentiated hepatocellular carcinoma. Cells form irregular cords and occasionally form canaliculi. Nuclei are vesicular with small vacuoles in some cells. H. and E. × 380. Insert shows more frequent vacuoles containing lipid. H. and E. × 340.

FIG. 9.—Poorly differentiated hepatocellular carcinoma. H. and E. × 500.

FIG. 10.—Undifferentiated hepatocellular carcinoma. The cells grow in sheets. They are small with little basophilic cytoplasm and round nuclei with single nucleoli. There are occasional mitotic figures. H. and E. × 380.

FIG. 11.—Highly differentiated hepatocellular carcinoma. Cells grow in sheets with obscure cord structure and sinusoids. Cytoplasm is eosinophilic and nuclei vesicular and sometimes double. H. and E. × 380.

FIG. 12. Highly differentiated hepatocellular carcinoma. Many of the cells contain glycogen within the cytoplasm PAS. ×380.

FIG. 13.—Poorly differentiated cholangiocellular carcinoma. The cells are columnar with lightly basophilic cytoplasm and basal oval shaped nuclei. They attempt to form glands or ducts in some parts. H. and E. × 320. Insert shows dense connective tissue observed in some areas of these carcinoma. H. and E. × 600.

FIG. 14.—Poorly differentiated cholangiocellular carcinoma. H. and E. × 500.

FIG. 15.—Well differentiated hepatocellular carcinoma. Hemosiderin, ceroid and bile are present in macrophages in focal strands of connective tissue. H. and E. × 320.

1 3

2

Reuber

4

6

5

Reuber

7

9

8

Reuber

10

11

12

13

14

15

The undifferentiated hepatocellular carcinoma had sheets of cells (Fig. 10). Nuclei were round with single nucleoli and cytoplasm was scanty and basophilic. Occasional cells had mitotic figures.

The highly differentiated hepatocellular carcinomas grew in sheets with obscure cord structure and sinusoids (Fig. 11). Nuclei were vesicular, sometimes double, and the cytoplasm was eosinophilic. Many of the cells contained glycogen within the cytoplasm (Fig. 12). The other carcinomas did not contain glycogen, except when highly differentiated were mixed with well-differentiated hepatocellular cells.

The poorly differentiated cholangiocellular cholangiocarcinoma was made up of spindle-shaped cells with oval nuclei and lightly basophilic cytoplasm (Fig. 13 and 14). There was a focal " desmoplastic " stromal reaction. The latter carcinomas have been described in detail previously (Reuber, 1967).

The only carcinoma with macrophages containing hemosiderin, ceroid and bile within bands of connective tissue was the well-differentiated hepatocellular carcinoma (Fig. 15).

Pulmonary metastases or metastases to other organs were not observed in any of the animals.

### TRANSPLANTS

The number of lesions transplanted and the number of lesions that grew are given in Table I.

TABLE I.—*Normal Liver and Pre-neoplastic and Neoplastic Lesions Transplanted**

| | Normal | Areas of hyperplasia | Nodules of hyperplasia | Small hepatocellular carcinomas | Large hepatocellular carcinomas |
|---|---|---|---|---|---|
| Autologous | 0/20 | 0/23 | 0/14 | — | 5/5 |
| Isologous | 0/49 | 0/53 | 0/42 | 2/3 | 20/22 |

* The denominator shows the number transplanted and the numerator the positive number.

*Autologous.*—Areas or nodules of hyperplasia (or normal liver) did not survive when transplanted subcutaneously into the same animal. Small carcinomas, because of the small number and the small amount of tissue available, were not transplanted into the same animal. Large carcinomas grew, but did not reach a very large size because the carcinomas developed late in the life of the animals, survival was not long enough, and growth rate of first generation transplants was slow. These carcinomas, however, grew well when later retransplanted into young animals isologously.

*Isologous.*—Areas and nodules of hyperplasia (or normal liver) did not survive when transplanted subcutaneously into animals of the same strain. Small and well developed carcinomas grew following transplantation to isologous hosts. Two of three small hepatocellular carcinomas (less than 5 mm. in diameter) grew on transplantation. The following well-developed carcinomas (greater than 5 mm.) survived and grew: 2 highly-differentiated, 3 well-differentiated, 2 highly- plus well-differentiated, 5 poorly differentiated carcinomas with eosinophilic cytoplasm, 2 well- plus poorly-differentiated, 2 poorly differentiated with basophilic cytoplasm, 1 poorly- plus undifferentiated, and 1 poorly differentiated cholangiocarcinoma. Two highly differentiated hepatocellular carcinomas did not grow. The carcinomas reached 6–7 cm. in greatest diameter and killed the hosts; however metastases were not observed. The histologic pattern of all transplantable carcinomas was the same as the primary carcinoma; however, in later generations the less malignant cells were lost in the carcinomas with 2 cell types.

The first transplant generations of the highly differentiated appeared between 8 and 12 months, the well-differentiated and poorly-differentiated with eosinophilic cytoplasm between 6 and 10 months, and the remaining carcinomas between 3 and 6 months.

The growth rate of all transplanted carcinomas increased in the second and later generations. The most notable change was observed with the poorly-differentiated carcinomas with eosinophilic cytoplasm. Their growth rate was similar to that of the poorly-differentiated carcinomas with basophilic cytoplasm, 2 to 4 months. Well-differentiated carcinomas grew in 3 to 6 months, and highly-differentiated 4 to 8 months in later generations.

### DISCUSSION

In the past, pathologists and biologists working and studying spontaneous or induced lesions of the liver in C3H, C3H × Y hybrid, and other strains of mice have not distinguished between hyperplasia and neoplasia. Andervont and Dunn (1952) concluded that all lesions of the liver regardless of size or morphology were neoplastic, and that there was no correlation between the transplantability and the morphologic pattern of hepatic lesions. The carcinomas in mice given carbon tetrachloride or o-aminoazotoluene often resembled lesions described here as hyperplastic (Andervont, 1958; Edwards, 1941; Edwards and Dalton, 1942; Edwards *et al.*, 1942; Eschenbrenner, 1944; and Eschenbrenner and Miller, 1946). Not unexpectedly, they usually did not grow on transplantation, except rarely in the spleen (Leduc and Wilson, 1959).

There was a close correlation between the morphologic and biologic behavior of hepatic lesions in C3H × Y hybrid mice in this study. Hyperplastic lesions did not survive or grow in autologous or isologous hosts. Carcinomas did grow and kill the host. Therefore, most of the lesions are hyperplastic and not carcinomas.

It would seem that the differences in the conclusions in this study and at least one previous study are related to the procedures. Hyperplastic nodules and carcinomas collide and on gross examination appear to be one large lesion (Reuber, 1965, 1966). If the tissue transplanted and that taken for section were chosen at random, neither would be representative. It is necessary to choose one lesion and to take tissue for transplantation and a section immediately adjacent for histologic study.

The results obtained on transplantation of hepatic lesions in mice are similar to those observed in rats ingesting N-2-fluorenyldiacetamide (Reuber and Firminger, 1963). There is a higher incidence of carcinomas and more well-differentiated carcinomas in the rats. Metastases of primary and transplantable carcinomas in rats often kill the host. Fewer large carcinomas are made up of smaller colliding lesions in the livers of rats with induced tumors compared to the spontaneous tumors in mice.

The only poorly differentiated cholangiocarcinoma in the present experiment that was transplanted grew. Previously only 8 of these carcinomas were described and it was felt that this carcinoma was rarely seen in mice (Reuber, 1967). However, since at present all hepatic tumors from each animal are being studied histologically, the incidence is much higher, as reported by Vlahakis and Heston (1971). These authors also described and illustrated a primary undifferentiated cholangiocarcinoma with pulmonary metastases. The carcinoma grew on transplantation.

Dunn and Andervont (1952, 1955) reported that transplanted hepatic carcinomas in mice developed into hemagiosarcomas, fibrosarcomas, reticulum cell sarcomas and even adenocarcinomas as early as the second generation. The histologic pattern of the carcinomas transplanted in this study have not changed, except for the more malignant cells overgrowing the less malignant, up to the fifteenth generation. The cells in poorly differentiated hepatocellular carcinomas also lost lipid vacuoles in the cytoplasm.

In conclusion, these results have shown that the transplantability of hyperplastic and neoplastic hepatic lesions occurring spontaneously in C3H $\times$ Y hybrid mice correlates with the histologic pattern. Based on these findings it is evident that most of the lesions are hyperplastic; few are carcinomas.

### REFERENCES

Andervont, H. B.—(1958) *J. natn. Cancer Inst.*, **20**, 431.

Andervont, H. B. and Dunn, T. B.—(1952) *J. natn. Cancer Inst.*, **13**, 455.

Dunn, T. B. and Andervont, H. V.—(1955) *J. natn. Cancer Inst.*, **15**, 1513.

Edwards, J. E.—(1941) *J. natn. Cancer Inst.*, **2**, 197.

Edwards, J. E. and Dalton, A. J.—(1942) *J. natn. Cancer Inst.*, **3**, 19.

Edwards, J. E., Heston, W. E. and Dalton, A. J.—(1942) *J. natn. Cancer Inst.*, **3**, 297.

Eschenbrenner, A. B.—(1944) *J. natn. Cancer Inst.*, **4**, 385.

Eschenbrenner, A. B. and Miller, E.—(1946) *J. natn. Cancer Inst.*, **6**, 325.

Heston, E. E.—(1966) *J. natn. Cancer Inst.*, **37**, 839.

Heston, W. E. and Vlahakis, G.—(1961) *J. natn. Cancer Inst.*, **26**, 969.

Heston, W. E., Vlahakis, G. and Deringer, M. K.—(1960) *J. natn Cancer Inst.*, **24**, 425.

Leduc, E. H. and Wilson, J. W.—(1959) *J. natn. Cancer Inst.*, **22**, 581.

Reuber, M. D.—(1965) *J. natn. Cancer Inst.*, **34**, 697.—(1966) *Gann Monogr.*, **1**, 43. —(1967) *J. natn. Cancer Inst.*, **38**, 901.

Reuber, M. D. and Firminger, H. I.—(1963) *J. natn. Cancer Inst.*, **31**, 1407.

Reuber, M. D. and Odashima, S.—(1967) *Gann*, **58**, 513.

Vlahakis, G. and Heston, W. E.—(1971) *J. natn. Cancer Inst.*, **46**, 677.